# Conservation Assessment and Chemistry of *Boswellia ogadensis,* a Critically Endangered Frankincense Tree

**DOI:** 10.3390/plants11233381

**Published:** 2022-12-05

**Authors:** Stephen Johnson, Abdinasir Abdikadir, Prabodh Satyal, Ambika Poudel, William N. Setzer

**Affiliations:** 1 FairSource Botanicals, LLC, 560 Fox Drive #643, Fox Island, WA 98333, USA; 2 Somali Region Pastoral and Agro-Pastoral Research Institute, Jigjiga P.O. Box 1020, Ethiopia; 3 The Aromatic Plant Research Center, 230 N 1200 E, Suite 100, Lehi, UT 84043, USA; 4 Department of Chemistry, University of Alabama in Huntsville, 301 Sparkman Drive, Huntsville, AL 35805-1911, USA

**Keywords:** *Boswellia ogadensis*, frankincense, Ethiopia, Ogaden, essential oil, endangered tree

## Abstract

*Boswellia ogadensis* is a critically endangered species of frankincense tree, restricted to a small area of the Shabelle river valley in southern Ethiopia. It has only been recorded from two botanical collecting trips, in 1972 and 2006, with no indication of the abundance, threats, or population status of the trees, and it was listed on the IUCN Red List of Endangered Species as “Critically Endangered” in 2018. More recent expeditions, in 2019 and 2021, were not able to locate the species, raising concerns about its continued survival. We carried out a field survey in June 2022 to re-locate the species, assess the threat level it is facing, and collect samples of resin for analysis. This survey revealed that *B. ogadensis* is present in more locations than previously recorded, and is more abundant than thought. While it is facing multiple threats, including grazing, cutting for firewood, and insect attacks, these threats vary geographically, and there are populations that appear to be healthy and regenerating well. While more research is needed, the current survey indicates that downlisting to “Endangered” status may be appropriate. Samples of resin were also collected and analyzed using gas chromatographic techniques, revealing that while the essential oil profile is similar to that of other *Boswellia* species (dominated by α-thujene, α-pinene, *p*-cymene, and terpenin-4-ol), there are chemical markers that can distinguish it from other sympatric *Boswellia* species, indicating the potential for this to be used as a tool to monitor whether *B. ogadensis* is being harvested alongside other more common *Boswellia* species.

## 1. Introduction

The genus *Boswellia* Roxb. ex Colebr. (Burseraceae: Sapindales) consists of approximately 24 species of small to medium trees, typically characterized by papery, exfoliating bark, imparipinnate leaves, and the production of aromatic resin via a deep red resiniferous layer of bark [1]. The genus is widely distributed across west and east Africa, southern Arabia, and the Indian subcontinent, with the Horn of Africa region featuring the highest species concentration, particularly Socotra Island, which hosts almost half (11 of 24) of known *Boswellia* species [1]. The taxonomy of *Boswellia* is still dynamic, with recent years seeing multiple species added or occasionally removed; genetic work is ongoing [1,2,3].

The genus is best known for its production of a highly aromatic, terpenoid oleo-gum-resin, called frankincense, which is produced and stored in resin canals in the bark [4,5]. The resin exudes whenever the bark is broken, either by an animal or insect, or intentionally by humans cutting the bark to extract the resin. Most frankincense has been widely used and traded around the world for thousands of years, and is considered to be one of the oldest internationally traded commodities [5,6]. It is prized for its use in traditional medicinal systems, such as Ayurveda and Traditional Chinese Medicine, as well as its use in religious ceremonies, cosmetics, and perfumery. Additionally, essential oils and extracts from frankincense resins have become increasingly popular, with several million kilograms of resin processed annually to meet these demands [5,7].

Many *Boswellia* species are facing significant sustainability challenges and probable or confirmed population declines due to a variety of factors [7,8,9,10,11,12]. Key threats to many species include grazing by ungulates (goats, cattle, camels), fire, land conversion for agriculture, improper or excessive harvesting of resin, and attacks by insects [7,8]. Grazing and fire kill seedlings or saplings, and in some cases can completely block the regeneration of the species [13]. Improper resin harvesting, often in combination with insect attacks, or land conversion kills adult trees and can result in the complete conversion of *Boswellia* woodlands. The combination of these threats can be significant; studies on *Boswellia papyrifera* in northern Ethiopia, Eritrea, and Sudan have projected wide-scale population reductions of more than 70% on average within 25 years [8]. Other studies have noted sustainability concerns in *B. sacra* and *B. frereana* in Somaliland [9] and Oman [14], *B. serrata* in India [12], and *B. elongata* and other *Boswellia* species on Socotra island [10,11]. While studies have primarily focused on the major commercial species—with the exception of several studies conducted on the endemic *Boswellia* on Socotra island—rarer species likely face similar pressures, but at a greater threat level due to their small population sizes and geographically restricted ranges.

*Boswellia ogadensis* Vollesen is one such unique frankincense species restricted only to gypsaceous hillsides in the Shabelle river valley in southeastern Ethiopia, where it grows with species of *Commiphora*, *Vachellia*, and *Senegalia* at elevations of 280–350 m above sea level [1]. It is known from only three locations, close together, on the road between Gode and Kelafo; the first specimen (used to describe the species) was taken in 1972 [15], while an expedition in 2006 found two additional sites where the tree was growing [1]. The species was assessed as “Critically Endangered B1ab(iii)” in 2018 [16], but subsequent collecting trips were unable to locate it in 2019 and 2021, raising further concern about its current status (personal communication with Boris Vrskovy and Sebsebe Demmisew). It is known to occur in mixed-use forests, where it could face a combination of pressures such as grazing and cutting for firewood or construction material. Additionally, resins from *Boswellia rivae* and other species are collected around the same area, and *B. ogadensis* resin may be collected and mixed in with the more common *B. rivae*, as has happened to other rare and endemic *Boswellia* species in Somaliland (*B. occulta* mixed in with *B. sacra* and *B. frereana*) [17] and India (*B. ovalifoliolata* substituted for *Commiphora wightii*) [18].

As a result of these concerns, a conservation survey was carried out in June 2022 to attempt to re-locate the species, confirm it is extant in the Shabelle River Valley, and assess the level of threat the species is facing. The study also aimed to collect a sample of resin, if possible, to identify potential chemical markers in *B. ogadensis* that could help determine if the species is being harvested and mixed in with other resins from the same region.

## 2. Results

### 2.1. Population Status of Boswellia ogadensis

**Figure 1:**
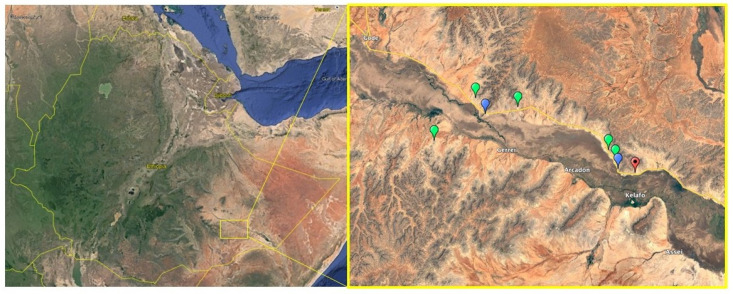
Map of locations where *B. ogadensis* occurs. Red pins are locations previously recorded where it was not found; blue pins are locations previously recorded where it was found; green pins are new locations not previously recorded.

*Boswellia ogadensis* was re-located at two out of the three previously known locations, and multiple additional populations along the Gode-Kelafo road were identified (Figure 1). A further population was found on the south bank of the Shabelle river valley, between the towns of Adadle and Gerrei (Jeerey), near the mountains known locally as Hul-Kujir. Although limited access prevented further exploration on the south bank, it is likely that additional populations exist in this area. The species was found only on gypsum hillsides, typically growing with *Commiphora guidottii* and other species of *Commiphora, Vachellia,* and *Senegalia,* but was abundant in all locations, with estimated densities of 50 trees or more per hectare commonplace. Although quantitative surveys will be required to definitively determine the total population size, these observations suggest it may be 10,000 mature individuals or more.

**Figure 2:**
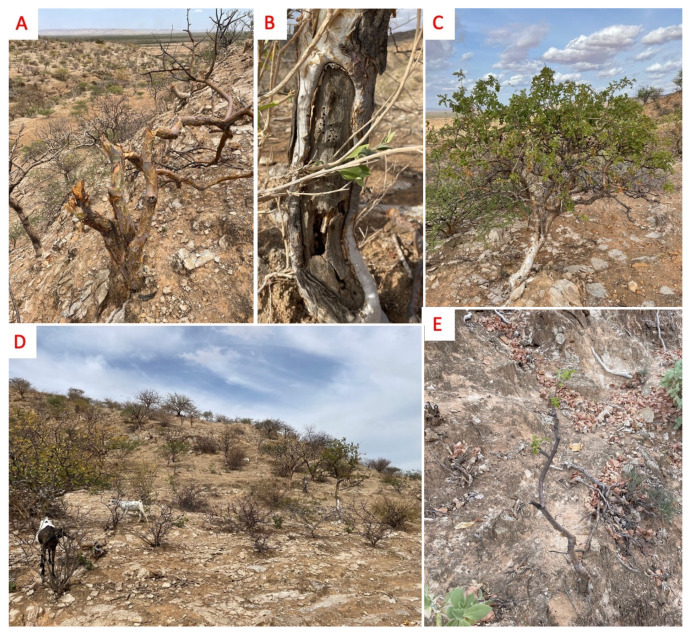
(**A**) Cut *B. ogadensis* tree; (**B**) Damage to *B. ogadensis* trunk showing insect attacks; (**C**) *B. ogadensis* individual; (**D**) Goats grazing on a hillside, with *B. ogadensis* visible in the background; (**E**) *B. ogadensis* sapling.

While the south bank population appeared to experience minimal anthropogenic disturbance, several pressures on the north bank populations were observed. Grazing by goats and sheep is evident in all populations, and villagers confirmed that animals graze in the hills. Very few young trees (<5 cm basal diameter) were observed in all but the south bank and western-most populations. Many trees in grazed populations also showed damage to the trunks of the trees, likely by grazing animals consuming the bark (see Figure 2). The damage to the trunks of the trees could be attempted resin harvesting rather than grazing damage, but active tapping is uncommon in this region, and villagers denied tapping the trees.

Multiple trees in one population had been cut down by humans, and in other populations cutting of other tree species was evident, suggesting this is relatively common practice but does not target *B. ogadensis* specifically. Evidence of attacks by boring insects, most likely cerambycid or buprestid beetles, was present in many populations as well. This seemed to primarily affect branches, with adult tree mortality due to insects rare. Mistletoes were also seen parasitizing trees in multiple populations. Erosion is likely a cause of natural mortality.

Without exception, villagers claimed little knowledge of *B. ogadensis.* Although many knew the tree, they refer to it as *mirafur*, the same name used for *B. rivae.* Only one villager identified it by a modified name, *mirafur silon* (“similar to *B. rivae* but different”). Many also said that they rarely visit the steep hillsides where *B. ogadensis* grows, although animals graze there.

### 2.2. Chemical Composition of Resin Samples

Resin samples were collected from naturally exuding trees in three locations. The resin essential oils were obtained by hydrodistillation in yields of 4.18% to 6.08% (w/w) as yellow oils. The resin essential oils were analyzed by gas chromatography—mass spectrometry (GC-MS), gas chromatography with flame ionization detection (GC-FID), and chiral GC-MS. The essential oil compositions are listed in Table 1 and the enantiomeric distribution of monoterpenoid components is presented in Table 2.

All three samples of the essential oil were dominated by monoterpenes, with almost no sesquiterpenes present. All three samples were rich in α-thujene (30.8–46.2%), *p*-cymene (9.0–14.5%), and terpenin-4-ol (5.4–14.8%), with sabinene (3.4–4.9%) and α-pinene (3.2–20.9%) present at lower levels, except in sample three, where it was the second most abundant component (20.9%) after α-thujene.

α-Thujene, camphene, α-thujone, and β-thujone were all found to be the dextrorotatory enantiomers exclusively. Sabinene showed a preponderance of (−)-sabinene (73.8–78.5%). Consistent with this, (−)-*cis*-sabinene hydrate was the dominant enantiomer (82.4–85.9%). α-Terpineol showed a variable mixture of enantiomers. The enantiomeric ratios for α-pinene, β-pinene, and limonene were also variable. (−)-Terpinen-4-ol was the predominant enantiomer, ranging from 81.7% to 92.4%.

## 3. Discussion

In this study, we aimed to re-locate and survey the previously recorded populations of *B. ogadensis* in the Shabelle river valley in southern Ethiopia, in order to assess the level of threat currently facing this unique species. Although the total range of the species is highly restricted, it was found to be locally abundant on the gypsaceous hills between Gode and Kelafo, and was found on the south bank of the river valley in addition to the north bank. The record from the south bank brings the total known Extent of Occurrence (EOO) to 405 km^2^, and the discovery of additional populations on the south bank—which seems highly likely—may expand this EOO potentially up to 1000 km^2^ or more. Furthermore, the south bank population appeared to be relatively undisturbed, with good regeneration and no obvious signs of anthropogenic disturbance. Given the expanded EOO, with an Area of Occurrence (AOO) well over 10 km^2^, observed differences in the south versus north bank populations, and larger than expected total population size, *B. ogadensis* may not be as threatened as previously assumed. Down listing from “Critically Endangered” (CR) to “Endangered” (EN) under the IUCN Red List Criteria [24] is likely warranted, but further quantitative research is needed, particularly on the south bank populations.

Like many other species of *Boswellia*, *B. ogadensis* is facing several threats. The most prominent is the grazing pressure, primarily by goats, which suppresses new seedlings and has been shown to completely block regeneration in other *Boswellia* species [8,11]. In this case, the grazing pressure varies geographically, with some sites containing almost no young trees and others containing evidence of robust regeneration. Unsurprisingly, populations near human settlements showed more intense grazing pressure while more remote sites were less grazed. The grazing can also cause damage to the trees’ bark, creating opportunities for boring insects to attack the tree. Boring beetles belonging to the Cerambycidae and Buprestidae families have been found to attack other *Boswellia* species, sometimes fatally [9,25,26]. However, few dead trees were observed, with most of the obvious insect damage occurring in branches or still-surviving trunks.

Cutting of trees, likely for firewood, is a threat. Cut *B. ogadensis* were observed in one population, and cutting of various other tree species was observed in other populations. Interestingly, local people did not identify the tree as having any distinct uses, and largely regarded it as equivalent to the far more common *B. rivae*. Although the cutting does not seem to target *B. ogadensis* specifically, the threat posed by general habitat degradation, particularly around villages, is still present.

The essential oil of *B. ogadensis* was revealed to be dominated by monoterpenes, particularly α-thujene, α-pinene, *p*-cymene, and terpenin-4-ol. This is similar to many other species of frankincense: *B. sacra* essential oil displays a variable chemical profile dominated by either α-pinene or more rarely α-thujene with inclusions of sabinene, myrcene, limonene, *p-*cymene, and other monoterpenes [27,28,29,30]; *B. frereana* essential oil is rich in both α-thujene and α-pinene, with sabinene and *p*-cymene [31]; and *B. serrata* essential oil is typically dominated by α-thujene with minor components including methyl chavicol, methyl eugenol, myrcene, sabinene, and kessane [32]. By contrast, other *Boswellia* species show unusual essential oil profiles, such as *B. papyrifera*, which is dominated by octyl acetate and octanol [33], or *B. occulta*, which is dominated by methoxyalkanes [34]. All three samples of *B. ogadensis* essential oil also included *trans-*sabinene hydrate acetate, which was previously suggested as a marker compound for *B. frereana* [35].

*Boswellia rivae*, *B. neglecta*, and *B. microphylla* all occur in the same geographic area as *B. ogadensis* [1]. *Boswellia rivae* essential oil is most often dominated by α-pinene, with limonene, δ-3-carene, *p*-cymene, and β-pinene often present as major components [36,37,38]. *Boswellia neglecta* essential oil is very similar to that of *B. ogadensis*, with high levels of α-thujene, α-pinene, *p*-cymene, and terpinen-4-ol [36,37,38]. The essential oil of *B. microphylla* has not been characterized. While similar to *B. neglecta* essential oil, the essential oils of *B. ogadensis* can be distinguished by the lack of linalyl acetate and the presence of 3,5-dimethoxytoluene. *Boswellia rivae* resin is also collected in the same area where *B. ogadensis* grows; however, *B. ogadensis* essential oil can be identified by the presence of 3,5-dimethoxytoluene, and the presence of (*Z*)-salvene and/or (*E*)-salvene.

The presence of positive markers (3,5-dimethoxytoluene and (*Z*)-salvene) that are present in all analyzed samples of *B. ogadensis*, but not present in other species commercially harvested in the same area, indicate the potential for these to be used as monitoring tools, to determine if commercial batches of *B. rivae* essential oil include the far rarer *B. ogadensis*.

There have been previous studies on the enantiomeric distribution of monoterpenoids in *Boswellia* essential oils. While (+)-α-thujene was the exclusive enantiomer in *B. ogadensis* essential oil, (−)-α-thujene was predominant in *B. carteri* [39,40] and *B. dalzielii* [41] essential oils. The major enantiomer in *B. sacra* was (+)-α-thujene [39]. α-Pinene showed variation in enantiomeric distribution in *B. ogadensis*, similar to those observed for *B. carteri* [39,40] and *B. dalzielii* [41] essential oils. In *B. carteri* [40] and *B. dalzielii* [41] essential oils, (−)-β-pinene predominated, in contrast to that found in *B. ogadensis*, which was nearly racemic. (−)-Sabinene was the predominant enantiomer in *B. ogadensis* essential oil, comparable to that found in *B. carteri* [39,40] and *B. dalzielii* [41] essential oils. (+)-Camphene was the exclusive enantiomer in *B. ogadensis* essential oil, consistent with that observed in *B. sacra* essential oil [39], but camphene was nearly racemic in *B. carteri* [39,40]. The enantiomers of limonene seem to be variable for *Boswellia* essential oils.

## 4. Materials and Methods

### 4.1. Field Surveying

Field surveys of possible *B. ogadensis* locations were conducted from the 5th–13th of June 2022. All previously known locations were visited and re-surveyed, and additional potential sites in the Shabelle river valley area that could host *B. ogadensis* were also visited. Where *B. ogadensis* was found, we estimated the number present, number of young trees (<5 cm basal diameter) present, health of trees, phenology, and any current or potential threats observed (cutting, resin harvesting, grazing, etc.). We also interviewed local communities in the area to determine whether they knew about the presence of *B. ogadensis*, if they had any specific name for it, and how they were using *B. ogadensis* and the broader ecosystem in which it is growing.

### 4.2. Collection of Resins

Resin was collected opportunistically from natural exudations from *B. ogadensis* trees; no trees were tapped or otherwise harmed to collect the resin. Only small amounts were exuding from individual trees, so we pooled the resin from multiple individual trees in the same location for each sample (see Table 3). The exuded resins collected were of varying ages, but we focused on collecting more recently exuded resins and excluded old, dry resins that had been on the tree for a long time. Resins collected were sealed in plastic bags and shipped to the Aromatic Plant Research Center for analysis. A voucher specimen of *B. ogadensis* was collected and deposited in the Jigjiga Herbarium at the Somali Region Pastoral and Agro-Pastoral Research Institute (specimen no. 7204). The *B. ogadensis* trees were identified by S.J. and A.A., following the description from [1], based on the characteristics: bark smooth and sometimes flaking; imparipinnate leaves, 5–9 foliate, 4–15 cm long, sparsely puberulous, with leaflets ovate-elliptic to subcircular; 3-locular, narrowly pyriform, glabrous fruits, 12–16 × 4.5–6 mm, and pyrenes 4–5 × 1.5–2 mm, narrowly trullate with a long-acuminate tip and short basal horn, and trigonous without wings.

### 4.3. Hydrodistillation of Resins

Hydrodistillations of the resin samples of *B. ogadensis* were carried out using Likens-Nickerson apparatus for 6 h to give yellow essential oils (see Table 3).

### 4.4. Gas Chromatographic-Mass Spectrometry

The *B. ogadensis* resins were analyzed by GC-MS with a Shimadzu GCMS-QP2010 Ultra (Shimadzu Scientific Instruments, Columbia, MD, USA) with ZB-5ms capillary column (Phenomenex, Torrance, CA, USA) as previously described [27]. Identification of the chemical components was carried out by comparison of the retention indices determined with respect to a homologous series of normal alkanes and our comparison of their mass spectra with those reported in the literature [20,21,22] and the Aromatic Plant Research Center’s in-house library [23]. A representative chromatogram is shown in Supplementary Figure S1.

### 4.5. Gas Chromatographic-Flame Ionization Detection

The *B. ogadensis* oleogum resin essential oils were analyzed by GC-FID using a Shimadzu GC 2010 (Shimadzu Scientific Instruments, Columbia, MD, USA) equipped with flame ionization detector, a split/splitless injector, and Shimadzu autosampler AOC-20i (Shimadzu Scientific Instruments, Columbia, MD, USA), with a ZB-5 capillary column (Phenomenex, Torrance, CA, USA) as previously described [27].

### 4.6. Chiral Gas Chromatographic-Mass Spectrometry

The *B. ogadensis* essential oils were analyzed by chiral GC-MS as previously reported [41]: Shimadzu GCMS-QP2010S instrument (Shimadzu Scientific Instruments, Columbia, MD, USA), Restek B-Dex 325 capillary column (30 m × 0.25 mm × 0.25 μm film) (Restek Corporation, Bellefonte, PA, USA). Enantiomers of monoterpenoids identified by comparison of retention times with authentic samples (Sigma-Aldrich, St. Louis, MO, USA) and percentages determined based on peak areas. A representative chiral gas chromatogram is shown in Supplementary Figure S2.

## 5. Conclusions

While restricted to a small range in the Shabelle river valley in southern Ethiopia, *Boswellia ogadensis* is more abundant than previously thought. The species is facing multiple threats, but some populations are regenerating well, and it does not appear to be specifically targeted by local people for firewood or resin harvesting. Given the expanded EOO and AOO, newly identified populations, and larger than expected total population size, *B. ogadensis* likely qualifies for down listing from Critically Endangered B1ab(iii) to Endangered B1 and B2ab(iii). The essential oil of *B. ogadensis* oleo-gum-resin is similar to that of other *Boswellia* species, but it can be distinguished from other sympatric *Boswellia* by the presence of 3,5-dimethoxytoluene and (*Z*)-salvene, which indicates the potential for use as an ex-situ monitoring tool. Despite the importance of this area as part of the Horn of Africa Biodiversity Hotspot, it is still under-studied and under-collected, with further research on the biodiversity, socio-economic importance, and drivers of land management needed. Further research is needed on *B. ogadensis* to identify additional populations and quantify current population structure and trends.

## Figures and Tables

**Table 1:** Chemical composition (%) of *Boswellia ogadensis* resin essential oils.

RT (Min)	RI_calc_	RI_db_	Compound	#1	#2	#3
5.965	778	766	Toluene	tr	tr	tr
8.403	846	846	(*Z*)-Salvene	0.2	0.2	0.2
8.746	855	856	(*E*)-Salvene	tr	0.1	tr
10.167	893	893	2-Bornene	0.1	0.1	0.1
10.778	905	902	Santolina triene	0.2	0.2	0.2
11.425	920	921	Hashishene	0.1	0.1	0.1
11.551	922	923	Tricyclene	---	---	0.1
11.841	927	925	α-Thujene	46.2	37.5	30.8
12.114	932	932	α-Pinene	3.2	6.9	20.9
12.591	941	943	Thujadiene	0.7	2.4	1.8
12.938	948	950	Camphene	0.2	0.5	1.1
13.138	951	953	Thuja-2,4(10)-diene	---	---	0.2
13.239	953	954	2,2-Dimethyl-5-methylenenorbornane	---	---	0.1
14.189	971	971	Sabinene	4.9	3.4	3.8
14.455	976	978	β-Pinene	0.2	0.4	1.3
14.785	982	982	6-Methyl-5-hepten-2-one	tr	tr	tr
15.070	987	989	Myrcene	tr	tr	tr
15.740	1000	1000	*p*-Menth-2-ene	---	0.1	---
16.096	1006	1006	α-Phellandrene	---	---	tr
16.156	1007	1009	2-Methylanisole	0.1	---	tr
16.613	1014	1015	1,4-Cineole	0.1	0.1	tr
16.743	1016	1017	α-Terpinene	0.2	0.4	0.4
16.910	1018	1022	*m*-Cymene	1.3	1.8	1.4
17.280	1024	1024	*p*-Cymene	9.0	14.5	11.4
17.397	1026	1026	2-Acetyl-3-methylfuran	2.8	2.2	1.9
17.523	1028	1030	Limonene	0.2	0.3	0.5
17.616	1029	1029	β-Phellandrene	tr	tr	tr
17.697	1030	1030	1,8-Cineole	0.1	0.1	tr
17.847	1033	1039	*o*-Cymene	---	---	0.1
17.912	1035	1036	3-Octen-2-one	tr	0.1	0.1
18.781	1048	---	Unidentified	1.2	1.0	0.8
19.333	1057	1057	γ-Terpinene	0.3	0.7	0.6
20.107	1069	1069	*cis*-Sabinene hydrate	0.4	0.3	0.2
20.235	1071	1071	*p*-Cresol	0.2	0.2	0.2
21.104	1084	1086	Terpinolene	0.1	0.1	0.2
21.417	1089	1091	*p*-Cymenene	0.1	0.1	0.1
22.144	1100	1101	*trans*-Sabinene hydrate	0.2	0.1	0.1
22.523	1106	1105	α-Thujone	0.2	0.3	0.2
22.950	1112	1112	2,4-Dimethyl-2,4-heptadienal	0.7	0.5	0.4
23.290	1117	1118	β-Thujone	2.1	2.2	1.8
23.365	1118	1118	Dehydrosabina ketone	0.1	0.1	0.1
23.724	1123	1124	*cis-p*-Menth-2-en-1-ol	0.1	0.2	0.1
23.880	1126	1126	α-Campholenal	---	---	0.2
24.762	1138	1138	*trans*-Sabinol	0.5	0.4	0.4
24.815	1139	1140	*trans*-Pinocarveol	---	0.1	0.4
24.950	1141	1139	*trans-p*-Menth-2-en-1-ol	0.1	0.1	---
24.923	1141	1141	*cis-*Verbenol	---	---	0.2
25.170	1145	1145	*trans*-Verbenol	0.1	0.2	1.2
25.503	1149	1150	α-Phellandren-8-ol	---	---	0.1
25.974	1157	1157	Sabina ketone	0.1	0.1	0.1
26.183	1160	1160	*trans*-Pinocamphone	---	---	0.1
26.293	1162	1164	Pinocarvone	---	---	0.1
26.825	1169	1169	Umbellulone	0.5	0.4	0.4
26.866	1170	1168	α-Phellandrene epoxide	1.0	1.0	0.9
27.033	1171	1171	*p*-Mentha-1,5-dien-8-ol	---	0.2	0.5
27.714	1182	1180	Terpinen-4-ol	14.8	12.4	5.4
27.790	1183	1183	Thuj-3-en-10-al	0.1	0.1	0.1
27.864	1185	1188	*p*-Methylacetophenone	0.1	0.3	0.2
28.022	1187	1186	*p*-Cymen-8-ol	0.8	1.6	1.4
28.360	1192	1194	*p*-Mentha-1,5-dien-7-ol	0.1	0.1	0.1
28.575	1195	1195	α-Terpineol	0.2	0.2	0.4
29.365	1206	1205	Verbenone	tr	0.1	0.5
29.490	1208	1208	*trans*-Piperitol	0.1	0.1	0.1
30.185	1218	1218	*trans*-Carveol	---	---	0.2
30.445	1223	1221	*p*-Cumenol	0.2	0.2	0.2
31.751	1241	1242	Cuminal	0.1	0.2	0.2
32.199	1248	1248	Carvotanacetone	0.2	0.6	0.5
32.306	1250	1258	*trans*-Sabinene hydrate acetate	0.2	0.2	0.2
32.688	1255	1257	Carvenone	0.1	0.1	---
33.319	1265	1265	3,5-Dimethoxytoluene	0.4	0.1	0.1
34.588	1283	1282	Bornyl acetate	0.1	0.2	0.6
34.997	1289	1289	Thymol	0.9	1.2	1.0
35.390	1295	1296	Terpinen-4-yl acetate	tr	---	---
35.536	1297	1300	Carvacrol	0.7	0.8	0.6
35.946	1303	1306	Isoascaridole	0.1	---	---
36.274	1308	1308	*cis*-2,3-Pinanediol	0.1	---	0.1
38.701	1345	1346	α-Terpinyl acetate	0.2	0.3	0.3
41.095	1382	1382	β-Bourbonene	0.1	0.2	0.2
			Monoterpene hydrocarbons	67.3	69.7	75.3
			Oxygenated monoterpenoids	24.5	24.3	19.3
			Sesquiterpene hydrocarbons	0.1	0.2	0.2
			Oxygenated sesquiterpenoids	---	---	---
			Benzenoid aromatics	0.7	0.6	0.6
			Others	3.5	2.9	2.4
			Total identified	96.0	97.6	97.8

RT = Retention time in minutes. RI_calc_ = Retention index determined with respect to a homologous series of *n*-alkanes on a ZB-5 ms column [19]. RI_db_ = Retention index from the databases [20,21,22,23]. #1, #2, #3 refer to the collection sites. --- = not detected. tr = trace (<0.05%).

**Table 2:** Enantiomeric distribution of monoterpenoid components in *Boswellia ogadensis* resin essential oils.

Compound	RT (Min)	Enantiomeric Distribution (%)		
		#1	#2	#3
(+)-α-Thujene ^a^	13.8	100	100	100
(+)-α-Pinene	16.3	39.77	42.6	93.94
(−)-α-Pinene	15.9	60.23	57.4	6.06
(+)-Camphene ^b^	18.4	100	100	100
(+)-Sabinene	19.8	26.16	22.7	21.49
(−)-Sabinene	20.6	73.84	77.26	78.51
(+)-β-Pinene	20.4	40.55	35.64	66.49
(−)-β-Pinene	20.9	59.44	64.36	33.51
(+)-Limonene	26.1	41.38	46.24	51.09
(−)-Limonene	25.5	58.62	53.75	48.91
(+)-cis-Sabinene hydrate	40.8	14.1	15.59	17.61
(−)-cis-Sabinene hydrate	41.4	85.9	84.41	82.39
(+)-α-Thujone ^c^	43.3	100	100	100
(+)-β-Thujone	46.0	100	100	100
(+)-Terpinen-4-ol	54.4	7.64	13.6	18.27
(−)-Terpinen-4-ol	54.8	92.36	86.4	81.73
(+)-α-Terpineol	60.6	38.11	39.22	60.61
(−)-α-Terpineol	59.7	61.89	60.78	39.39

^a^ Due to the retention time proximity (13.8 min for (+)-α-thujene and 14.0 min for (−)-α-thujene) and the width of the peak, contribution of the other enantiomer cannot be ruled out. ^b^ Retention time for (−)-camphene = 17.7 min. ^c^ Retention time for (−)-α-thujone = 44.9 min.

**Table 3:** *Boswellia ogadensis* collection and hydrodistillation details.

Collection Site	Mass Resin	Mass Essential Oil (Yield)
Site #1: 05°46.00’ N, 43°51.00′ E, 434 m asl	34.44 g	2.0948 g (6.08%)
Site #2: 05°42.05′ N, 43°44.21′ E, 415 m asl	30.20 g	1.2629 g (4.18%)
Site #3: 05°41.35′ N, 44°08.15′ E, 369 m asl	23.07 g	1.2977 g (5.63%)

## Data Availability

Data are available from the corresponding authors (S.J. or W.N.S.) upon reasonable request.

## Supplementary Materials

The following supporting information can be downloaded at: https://www.mdpi.com/article/10.3390/plants11233381/s1, Figure S1: Gas chromatograms of *Boswellia ogadensis* oleogum resin essential oils; Figure S2: Chiral gas chromatogram of *Boswellia ogadensis* oleogum resin essential oil.
