# Supplementary material for: Conservation Assessment and Chemistry of Boswellia ogadensis, a Critically Endangered Frankincense Tree
Source: Plants (Basel). 2022 Dec 5;11(23):3381. doi: 10.3390/plants11233381 (PMC9735944; doi:10.3390/plants11233381)
Supplement: Supplementary file 1 [file plants-11-03381-s001.zip › plants-2030998-Supplementary/plants-2030998-Figure S1.pdf]

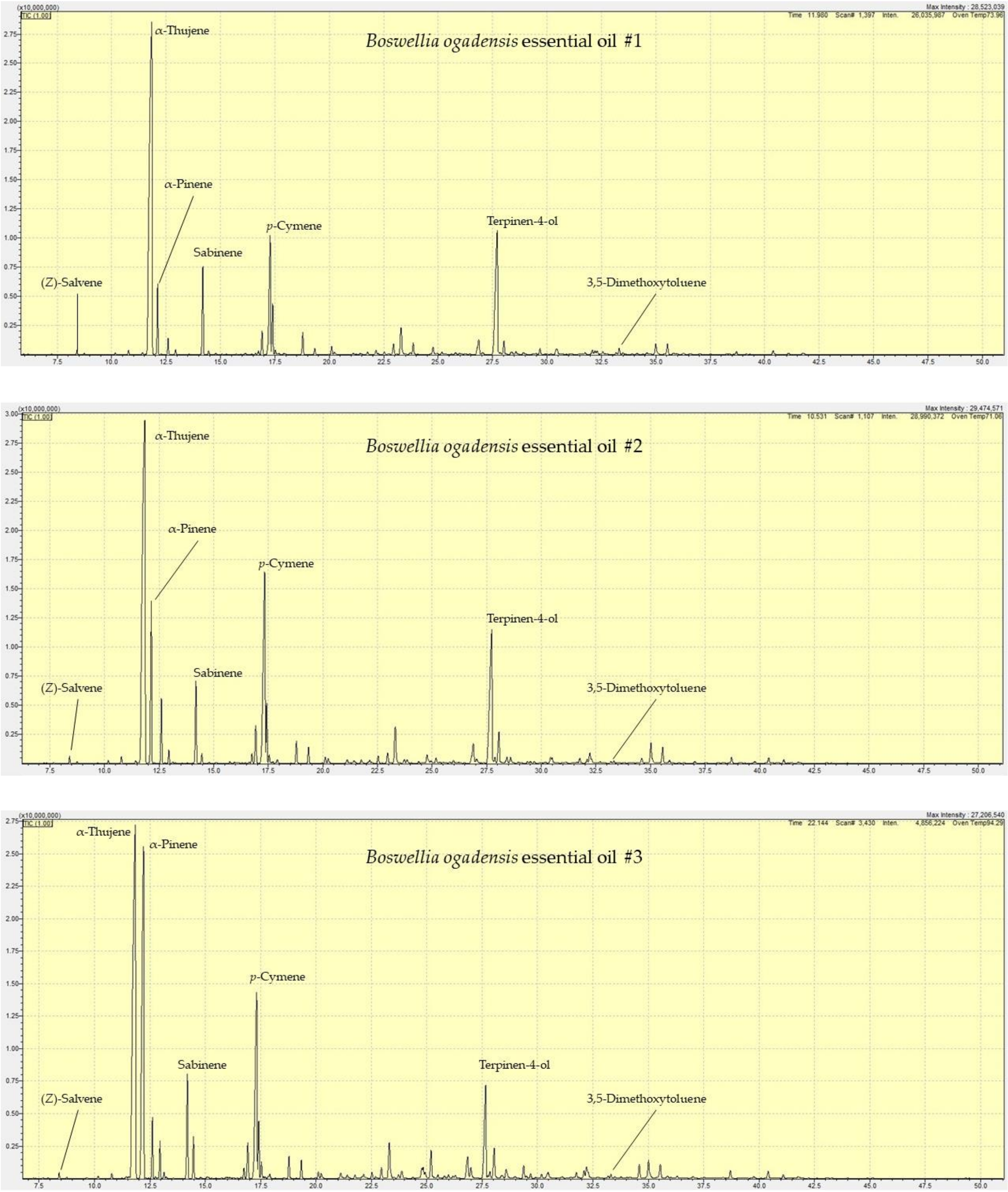

Supplementary Figure S1. Gas chromatograms of *Boswellia ogadensis* oleogum resin essential oils.
