# Supplementary material for: Conservation Assessment and Chemistry of Boswellia ogadensis, a Critically Endangered Frankincense Tree
Source: Plants (Basel). 2022 Dec 5;11(23):3381. doi: 10.3390/plants11233381 (PMC9735944; doi:10.3390/plants11233381)
Supplement: Supplementary file 1 [file plants-11-03381-s001.zip › plants-2030998-Supplementary/plants-2030998-Figure S2.pdf]

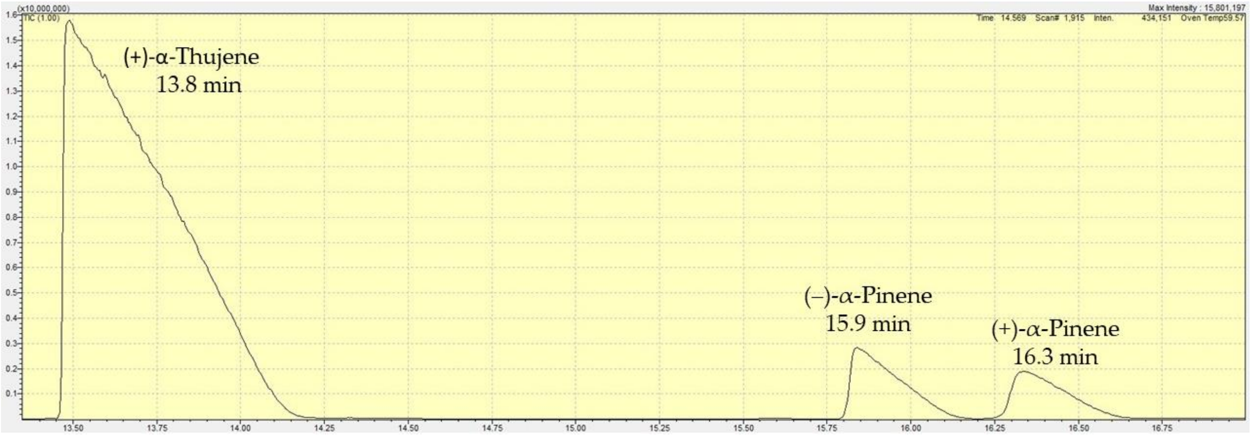
(+)-α-Thujene
13.8 min
(−)-α-Pinene
15.9 min
(+)-α-Pinene
16.3 min

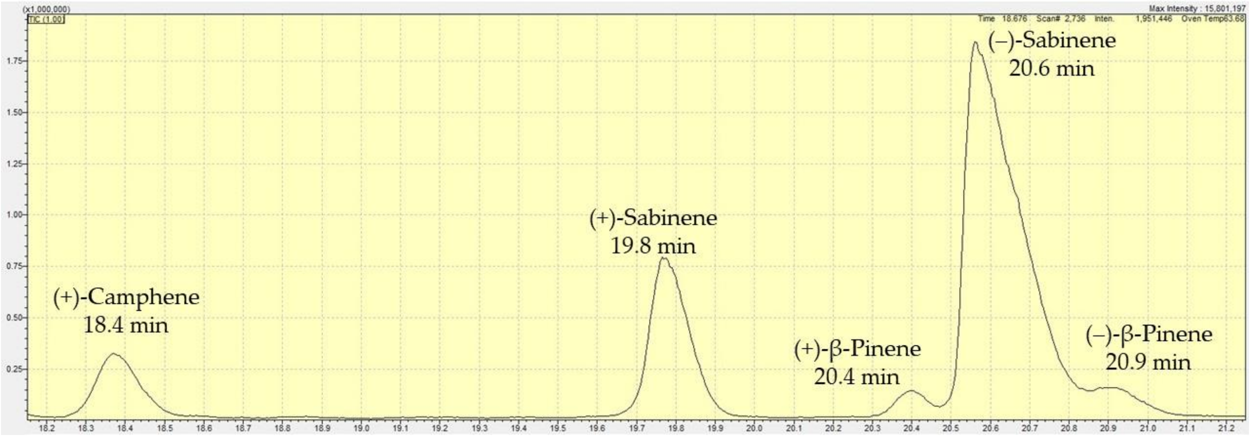
(−)-Sabinene
20.6 min
(+)-Sabinene
19.8 min
(+)-Camphene
18.4 min
(+)-β-Pinene
20.4 min
(−)-β-Pinene
20.9 min

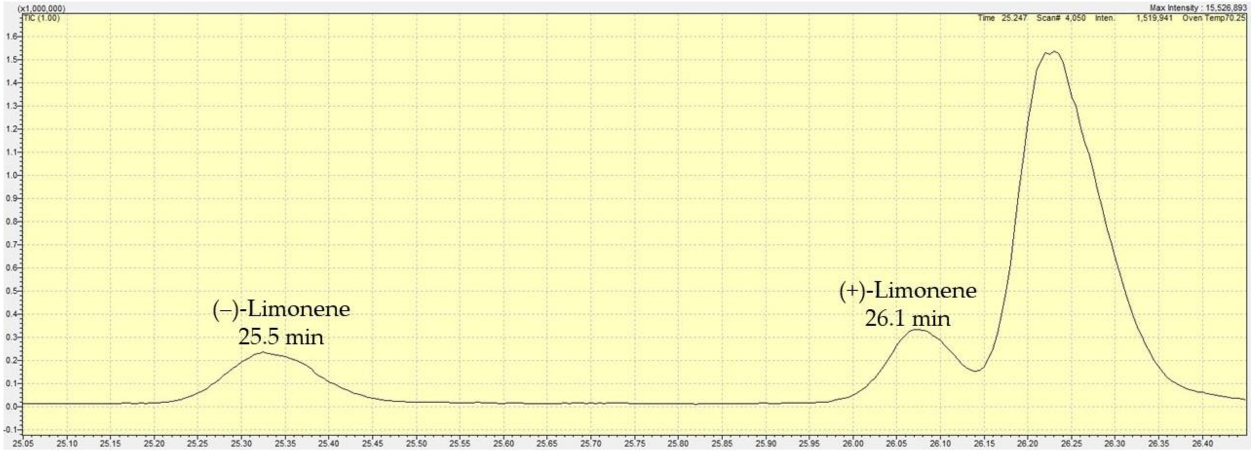
(+)-Limonene
26.1 min
(−)-Limonene
25.5 min

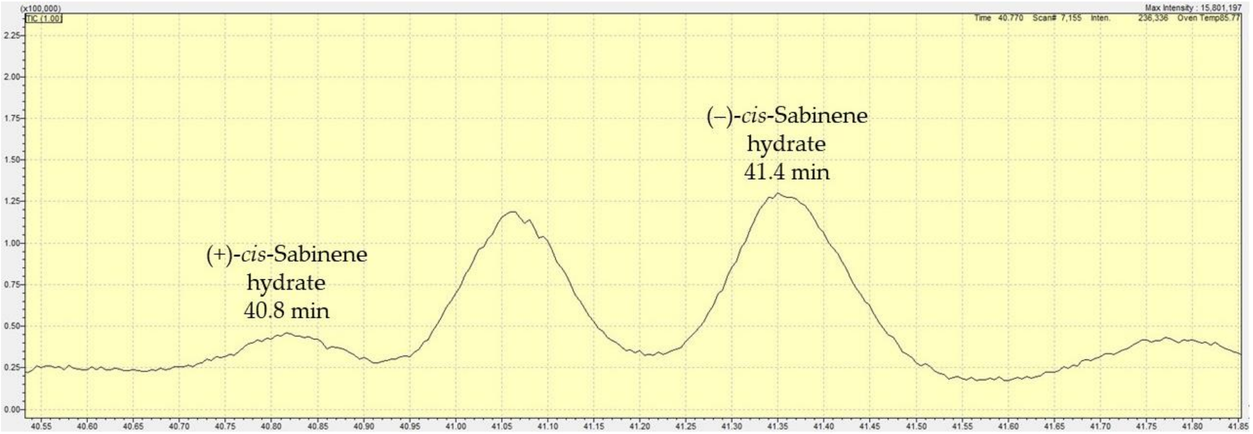
(–)-*cis*-Sabinene hydrate 41.4 min
(+)-*cis*-Sabinene hydrate 40.8 min

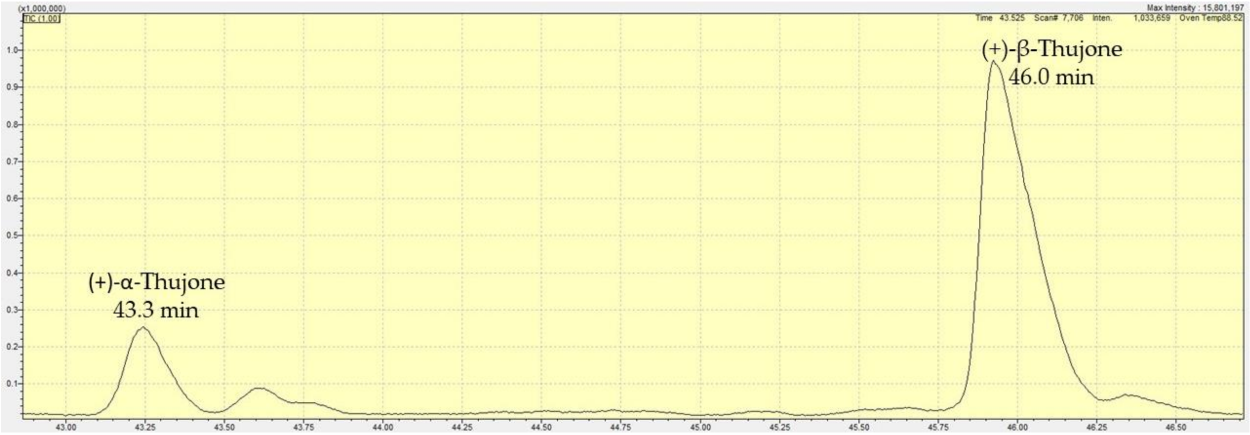
(+)-β-Thujone 46.0 min
(+)-α-Thujone 43.3 min

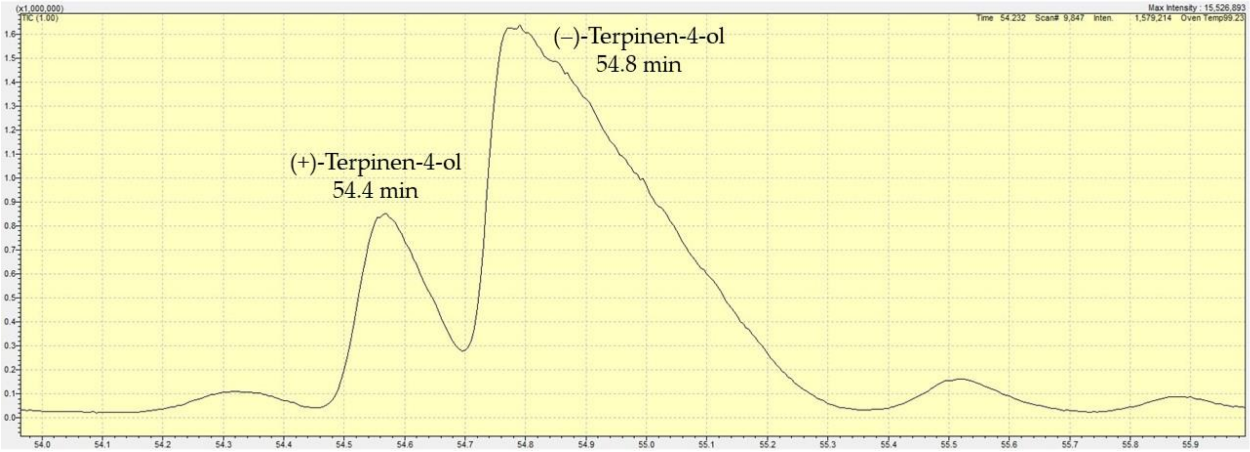
(–)-Terpinen-4-ol 54.8 min
(+)-Terpinen-4-ol 54.4 min

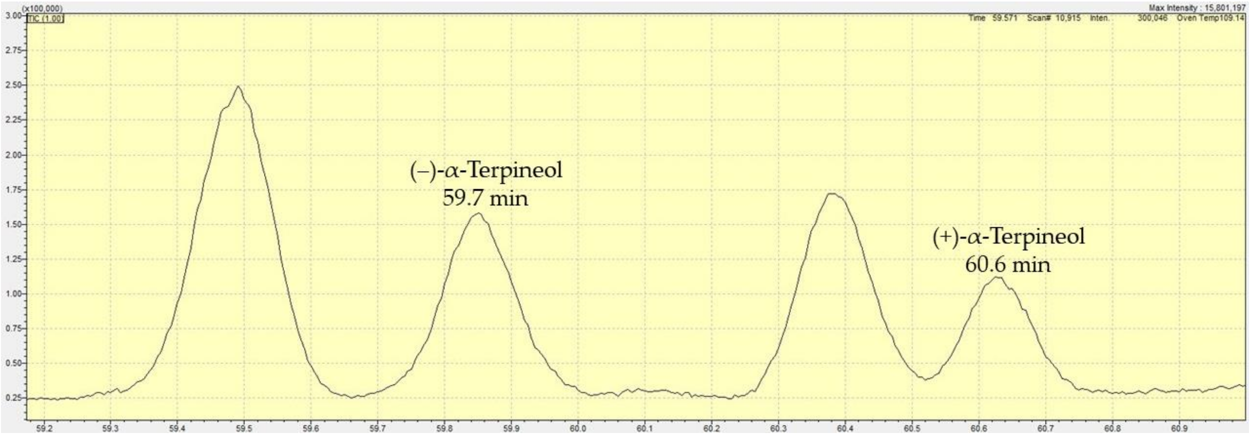

Supplementary Figure S2. Chiral gas chromatogram of *Boswellia ogadensis* oleogum resin essential oil.
